# Radiotoxicity in robotic radiosurgery: proposing a new quality index for optimizing the treatment planning of brain metastases

**DOI:** 10.1186/s13014-017-0867-z

**Published:** 2017-08-17

**Authors:** Alexandra Hellerbach, Klaus Luyken, Mauritius Hoevels, Andreas Gierich, Daniel Rueß, Wolfgang W. Baus, Martin Kocher, Maximilian I. Ruge, Harald Treuer

**Affiliations:** 1 Department of Stereotaxy and Functional Neurosurgery, University Hospital Cologne, Cologne, Germany; 2 Department of Radiotherapy, University Hospital Cologne, Cologne, Germany

**Keywords:** Stereotactic radiosurgery, Robotic radiosurgery, Brain metastases, Radiotoxicity, Dosimetry, Dose fall-off prediction

## Abstract

**Background:**

As irradiated brain volume at 12 Gy (V12) is a predictor for radionecrosis, the purpose of the study was to develop a model for Cyberknife (CK) plans that is able to predict the lowest achievable V12 at a given tumor size and prescription dose (PD), and to suggest a new quality index regarding V12 for optimizing the treatment planning of brain metastases.

**Method:**

In our model V12 was approximated as a spherical shell around the tumor volume. The radial distance between tumor surface and the 12 Gy isodose line was calculated using an approximation of the mean dose gradient in that area. Assuming a radially symmetrical irradiation from the upper half space, the dose distribution is given by the superposition of single fields. The dose profiles of a single field were derived by the measured off-center ratios (OCR) of the CK system. Using the calculated gradients of the sum dose profiles, minimal-V12 was estimated for different tumor sizes. The model calculation was tested using a phantom dataset and retrospectively applied on clinical cases.

**Results:**

Our model allows the prediction of a best-case scenario for V12 at a given tumor size and PD which was confirmed by the results of the isocentric phantom plans. The results of the non-isocentric phantom plans showed that an optimization of coverage caused an increase in V12. This was in accordance with the results of the retrospective analysis. V12 s of the clinical cases were on average twice that of the predicted model calculation. A good agreement was achieved for plans with an optimal conformity index (nCI). Re-planning of cases with high V12 showed that lower values could be reached by selecting smaller collimators and by allowing a larger number of total MU and more MU per beam.

**Conclusions:**

V12 is a main parameter for assessing plan quality in terms of radiotoxicity. The index f12 defined as the ratio of V12 from the actual plan with the evaluated V12 from our model describes the conformity of an optimally possible V12 and thus can be used as a new quality index for optimizing treatment plans.

**Electronic supplementary material:**

The online version of this article (doi:10.1186/s13014-017-0867-z) contains supplementary material, which is available to authorized users.

## Background

Stereotactic radiosurgery (SRS) is an established and widely used technique for the treatment of single and multiple brain metastases [1–3]. For the treatment of brain metastases, different radiosurgical modalities such as gamma knife (GK), LINAC-SRS or the robotic Cyberknife system (CK) exist, which all show dosimetrically comparable plan quality [4–7]. CK (Accuray, Sunnyvale, CA, USA) has the advantage of a frameless and image-guided targeting technology, which not only enables whole-body radiosurgery, but also hypofractionated treatment of large tumors (>3 cm) [8, 9]. The concept of hypofractionated treatment and the possibility to treat patients with up to ten brain metastases without whole-brain radiotherapy [10] underlines the attractiveness of SRS as a minimal-invasive, effective and safe therapy option for brain metastases. Nevertheless, side effects may arise whereby the development of brain radionecrosis represents the most common complication after SRS [11–16].

The risk of radionecrosis increases with larger doses and sizes of the target volume. Besides risk factors such as tumor biology [17], the volume of healthy brain tissue that is irradiated with a single dose of more than 10 Gy (V10) or 12 Gy (V12) is a predictor of brain radionecrosis [11, 12, 18–21]. Blonigen et al. and Minniti et al. have shown that the risk of radionecrosis increases gradually with V10 and V12 [20, 21]. Bohoudi et al. have presented a formula for estimating the optimal individual radiosurgery dose for isotoxic treatment planning of brain metastases [22]. In our study we did not aim to optimize the prescription dose in order not to exceed a maximum accepted V12. The objective of our study was to develop a model that allows the prediction of a minimal achievable V12 for the treatment of brain metastases with the CK system. Based on the presented model we suggest a new quality parameter which can be used for assessing V12 in the context of other plan quality criterions.

## Methods

### Derivation of a model for estimating a minimal-V12

Physical and geometrical assumptions have been made to develop a predictive model for estimating a minimal-V12 at a given tumor volume and prescription dose (PD). One assumption is that all metastases are spherical and that the prescription isodose-line can be fitted perfectly to the target surface. Thereby V12 is given as spherical shell around the planning tumor volume (PTV). For singular metastases V12 can be evaluated as follows:

1 $$ V{12}_{sing}(PTV)=\frac{4}{3}\pi {\left({r}_{PTV}+\varDelta {r}_{12}\right)}^3- PTV $$

where Δr_12_ is the radial distance between the tumor surface and the 12 Gy isodose-line. It depends on the prescribed dose and steepness of the dose gradient $\overrightarrow{\nabla}D$ and can be derived using an approximation of the mean dose gradient $\left\langle \left|\overrightarrow{\nabla}D\right|\right\rangle$ between the tumor surface and the 12 Gy isodose-line:

2 $$ \varDelta {r}_{12}=\frac{\varDelta D}{\left\langle \left|\overrightarrow{\nabla}D\right|\right\rangle}\kern0.5em \mathrm{with}\kern0.5em \overrightarrow{\nabla}D=\frac{\partial D}{\partial r}{\hat{e}}_r $$

**Fig. 1:**
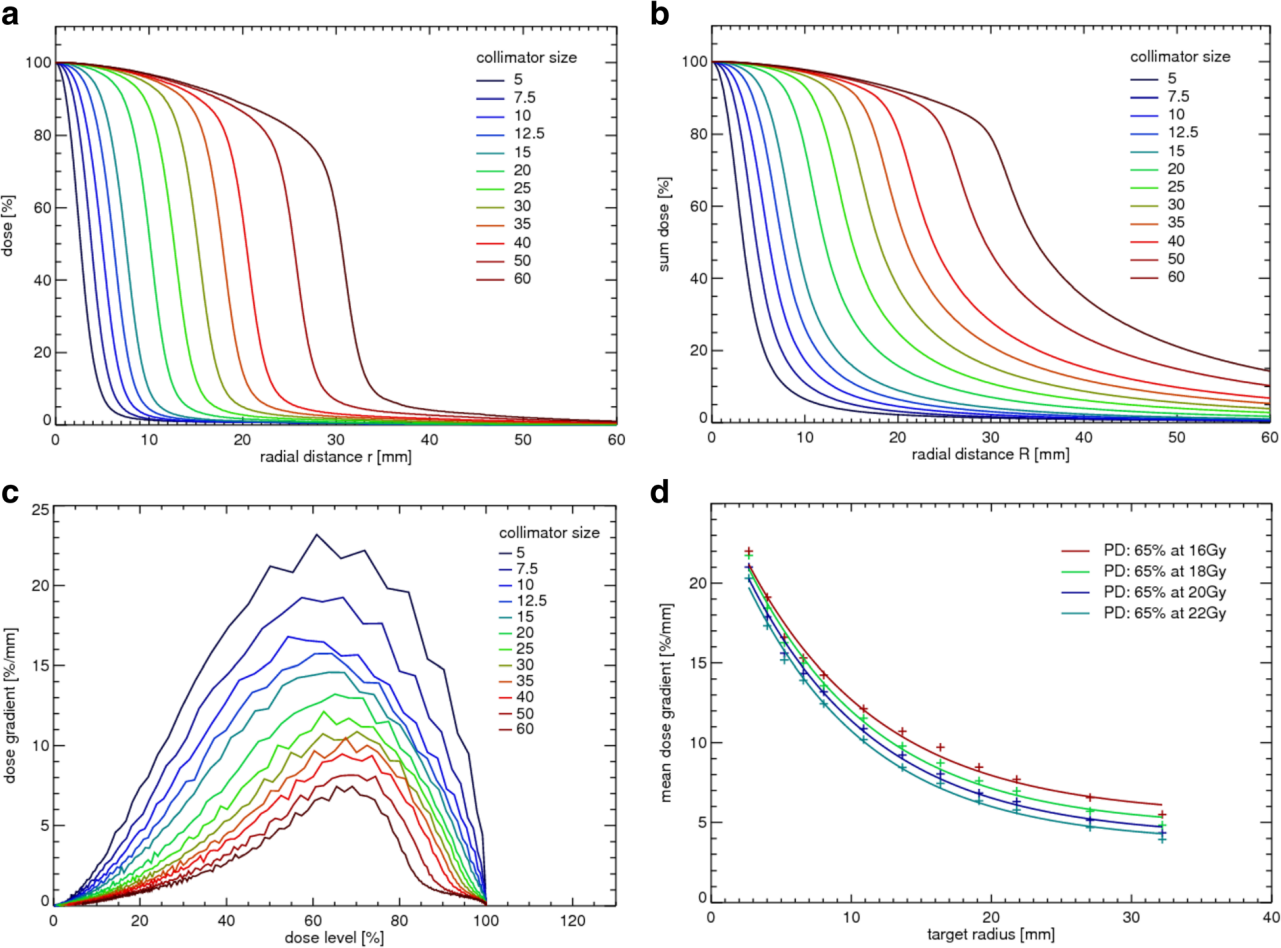
Derivation of the V12 model **a**: Measured OCR-curves for the 12 available collimator sizes of the CK system at a depth of 10 cm and 80 cm source-detector distance, r is the radial distance of the central axis. **b**: Sum dose profiles calculated by Eq. 3 where R represents the radial distance of the target center. **c**: Gradient of sum dose profiles calculated by numerical differentiation. **d**: Mean values of the dose gradient (in the range between 12 Gy and PD) are plotted as a function of the target radius (approximated by radial distance R of the sum dose profiles at 65%)

ΔD describes the dose difference between PD and 12 Gy. For the approximation of the mean dose gradient the measured off-center ratios (OCR) of the CK at a depth of 10 cm with an 80 cm source-detector distance have been used (Fig. 1a). Assuming an isocentric irradiation technique from the upper half space of a sphere that represents the possible beam setup for the CK system, the dose distribution of a spherical metastasis is given by the superposition of single fields, which summed up to a cumulative dose profile [23]:

3 $$ D(R)=2\pi {\int}_0^{\pi /2} d\theta \kern0.5em sin\theta \kern0.5em OCR\left(R\  sin\theta \right) $$

The radial distance R is given by r/sinθ where r is the radial distance of the central axis. All single fields were equally weighted, the depth-dose has been neglected and an optimal coverage of the PTV has been expected. A program for dose calculation was developed and implemented using IDL (Exelis Visual Information Solutions, Boulder, CO, USA). The sum dose profiles were calculated for different collimator sizes using Eq. 3 (Fig. 1b) and the corresponding dose gradients were determined for all dose profiles by numerical differentiation (Fig. 1c). Assuming the availability of a continuously adjustable collimator that would be able to precisely cover any tumor size with optimum conformity, it is possible to find a fit formula which describes the dose fall-off of any desired tumor size (Fig. 1d):

4 $$ \left\langle \left|\overrightarrow{\nabla}D\right|\right\rangle ={A}^{\ast}\exp \left({b}^{\ast }R\right)+{y}_0 $$

Using Eqs. 4 and 2 minimal-V12 can be calculated for any desired tumor volume at a given PD and can be compared to that of the CK plan.

For multiple metastases the total minimal-V12 can be derived as the sum of the calculated V12 s (Eq. 1) from each individual metastasis:

5 $$ V{12}_{mult,1}={\sum}_iV{12}_{i, sing}\left({PTV}_i\right) $$

Another possibility would be only considering the total target volume so that the total minimal-V12 is given by:

6 $$ V{12}_{mult,2}=V{12}_{sing}\left({\sum}_i{PTV}_i\right) $$

### Model validation and application

**Fig. 2:**
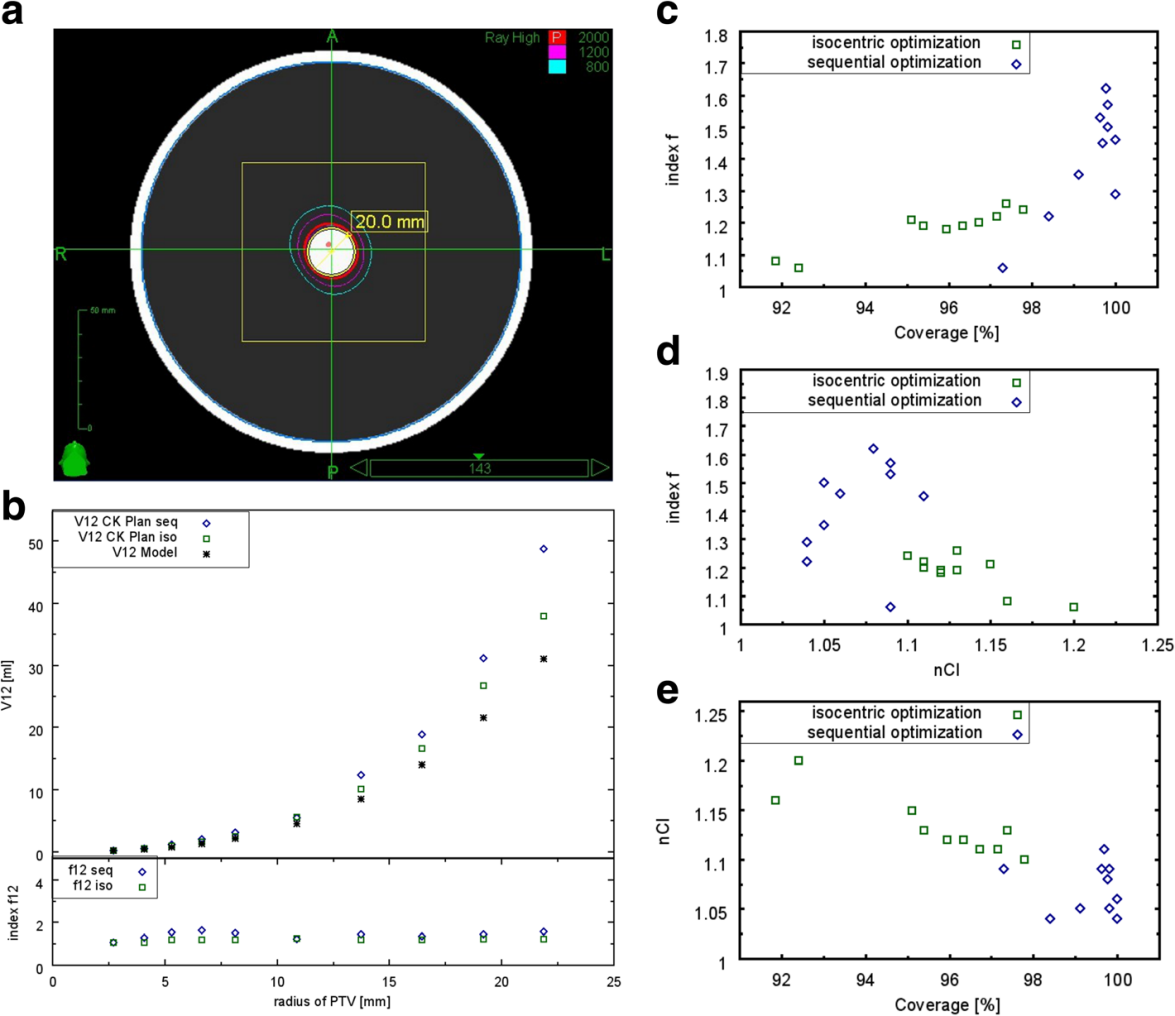
Model validation (**a**): Example of an isocentric phantom plan using the 20 mm collimator. **b**: Comparison of the resulting V12 s of isocentric and non-isocentric CK plans with the predicted V12 s of the model for ten different collimator sizes (5 mm - 40 mm). Relationship between (**c**): index f12 and coverage, **d**: index f12 and nCI, **e**: coverage and nCI

A set of virtual CT phantoms, created with MATLAB (MathWorks, Version 9.0) were used for model validation. The phantoms comprise a spherical volume filled with water (HU = 0) and embedded in a bone shell (HU = 1000). The Hounsfield values outside the sphere were set to −1000 (air). Spherical tumors of different size were set in the centre of each phantom. For ten CK collimators (diameter 5 up to 40) an optimal target radius using the radial distance R of the sum dose profiles at 65% (see Fig. 1b) was used for generating the spherical tumors. To simulate treatment cases as close as possible to our model assumptions, isocentric CK plans of the phantom dataset were performed using MultiPlan (Accuray, Version 4.5) (Fig. 2a). A PD of 65% at 20 Gy, commonly used in our department for the treatment of brain metastases, was applied. V12 s of CK plans were compared with the calculated minimal-V12 of our model. Additionally we re-planned all phantom cases using the sequential planning option of MultiPlan. We chose the same optimization planning strategy as for our clinical cases to analyze the effect of the non-isocentric irradiation technique on V12 compared to our model. To assess the application of our model on clinical cases, we analyzed retrospectively a patient collective comprising the treatment of 176 metastases and 67 patients. The patient collective was chosen randomly (in alphabetical order) from our data pool. The planning strategy of the sequential optimization included coverage (Cov = PTV covered by the prescription isodose volume (PIV)/PTV), three shells for optimizing conformity (CI = Cov * (PTV covered by the PIV)/PIV) [24, 25] and minimal monitor units (MU) as inverse planning objectives. The collimator selection was made manually for each case. Several collimator settings were tested to find the best solution in terms of dosage conformity, minimal dose in target volume, coverage and V10 and V12, respectively. Beam reduction was performed to allow a minimum MU of 10 per beam. MU limits for maximum MU per beam varied between 150 and 200 depending on PTV size and number of metastases. All treatments were performed in one fraction. The deviation of the predicted V12 s from the resulting V12 s of the CK plans was described by index f12, which is given by the following ratio:

7 $$ f12=\frac{V{12}_{CK\_ plan}}{V{12}_{model}} $$

The influence of two calculation methods (Eq. 5 versus Eq. 6) on V12 estimation for multiple metastases was compared and the dependence of index f12 on different factors was analyzed in terms of target volume, tumor location, number of metastases, new conformity index (nCI = 1/CI) [26], treatment time and total number of MUs.

## Results

### Model derivation and validation

The calculated sum dose profiles for the 12 existing collimator sizes showed that the slope of the dose profiles varies depending on the field size (Fig. 1b). Small field sizes showed sharp dose gradients whereas larger field size gradients were flatter. The steepest dose gradient was in the range of 55% - 65% for collimator diameters between 5 and 15 mm and drifted to higher dose levels (65% - 70%) for collimator diameters between 20 and 60 mm (Fig. 1c). The mean dose gradient decreased exponentially with increasing PTV radius (Fig. 1d). Results of the exponential fits (cf. equation 4) are shown in Table 1.

**Table 1:** Fit results for the four prescription doses used in our study

	65% at 16 Gy	65% at 18 Gy	65% at 20 Gy	65% at 22 Gy
A	20.913	21.732	21.692	21.666
b	−0.105	−0.109	−0.109	−0.113
y_0_	5.397	4.680	4.067	3.715
R^2^	0.991	0.993	0.995	0.997

$\left\langle \left|\overrightarrow{\nabla}D\right|\right\rangle$ is given by A*exp.(b*R) + y_0_. R^2^ is the coefficient of determination

Resulting V12 s of the isocentric plans in the phantom dataset showed a good agreement with the predicted V12 s of our model (Fig. 2b). Index f12 was independent on tumor size for both irradiation techniques. For isocentric plans, f12 ranged from 1.06 to 1.26 (mean = 1.18 ± 0.06) and showed no dependence on coverage (mean = 95.6% ± 2.0%, range: 91.9% - 97.8%) and nCI (mean = 1.13 ± 0.03, range: 1.10–1.20) (Fig. 2c, d). The sequential optimization yielded results with better coverage (mean = 99.4% ± 0.9%, range: 97.3% - 100%) and lower nCI (mean = 1.07 ± 0.02, range: 1.04–1.11) compared to isocentric plans, but at the cost of larger f12 values (mean = 1.41 ± 0.17, range: 1.06–1.62). Index f12 and coverage showed an opposite trend while using the sequential optimization technique, whereas in terms of nCI no trend was observable (Fig. 2c, d). A better coverage is per definition associated with a lower nCI; this effect was dominant for isocentric plans, whereas due to the sequential optimization technique both coverage and conformity were on a high level for non-isocentric plans (Fig. 2e).

### Model application on patient data

The retrospective analysis of the patient collective included 40 cases of singular and 40 cases of multiple metastases (176 metastases in total). The number of metastases per treatment ranged from 1 to 7 and was on average 2.2. In 72 cases PD was 65% at 20 Gy, in 4 cases 65% at 18 Gy, in 3 cases 65% at 22 Gy and in one case 65% at 16 Gy. The total PTV per treatment ranged from 0.09 ml to 46.07 ml and was on average 4.47 ml. The PTV per metastasis ranged from 0.04 ml to 39.88 ml and was on average 1.78 ml. The mean coverage of the CK plans of the patient data was 99.4% ± 1.2% (range: 92.1% to 100%) and the treatment time per metastasis was less than 60 min.

**Fig. 3:**
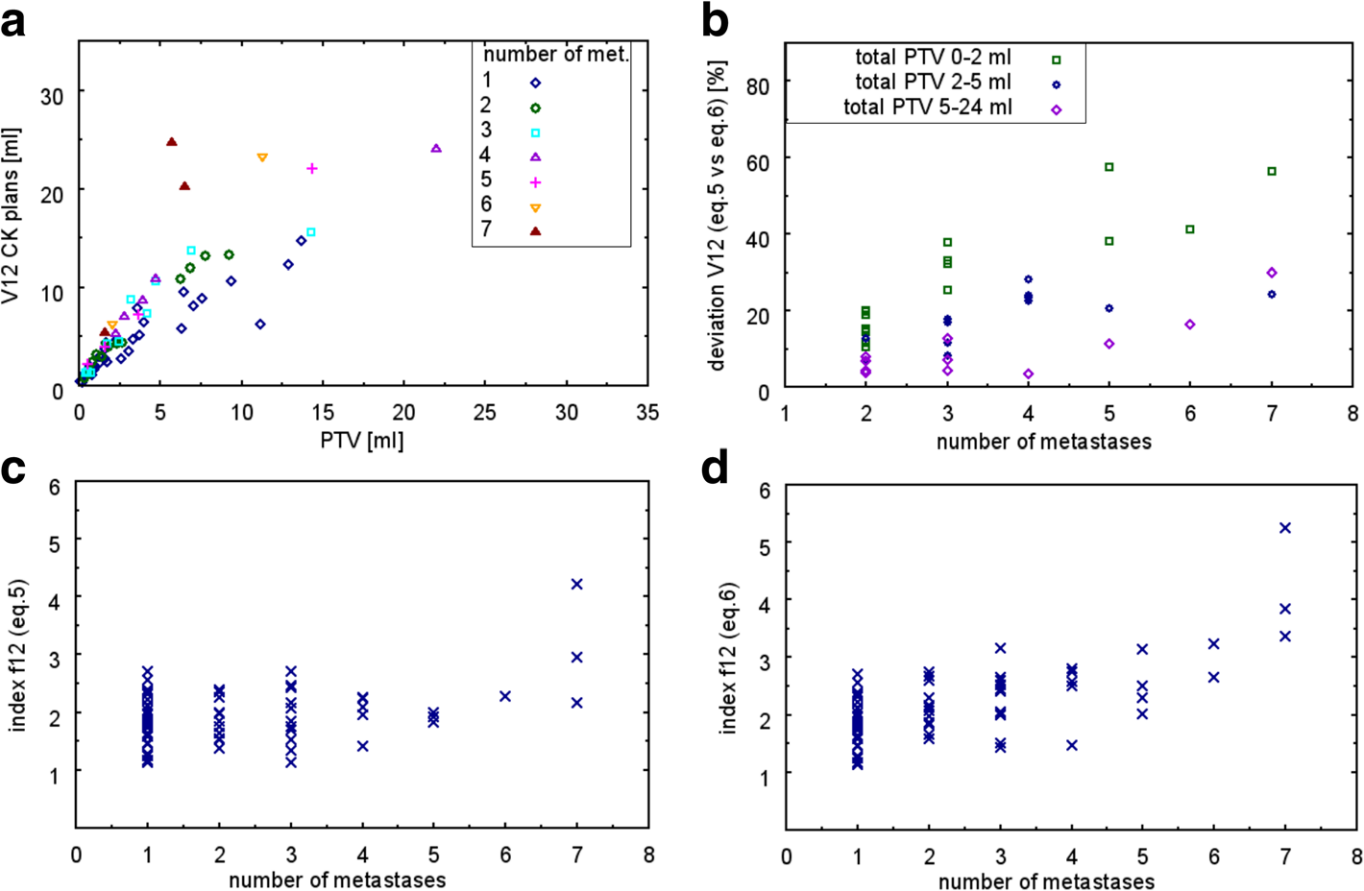
Influence of multiple metastases on V12 (**a**): Resulting V12 s of the non-isocentric CK plans, plotted as a function of total PTV subdivided into the number of metastases for a subset of data (all treatments with total PTV < 30 ml, includes 78 of 80 treatments). **b**: Deviation of Eq. 5 versus Eq. 6 for V12 estimation of multiple metastases. (**c)**: Index f12 depending on the number of metastases calculated by Eq. 5 and in (**d**): calculated by Eq. 6

Our data showed that the resulting V12 s of the CK plans are influenced by the number of metastases. Cases that had a similar total target volume but different number of metastases showed V12 increasing with the number of metastases (Fig. 3a). For V12 determination of multiple metastases, two calculation options (Eq. 5 versus Eq. 6) are possible which causes different results (Fig. 3b). The deviation between the two calculation methods was on average 19.5% ± 13.3% (range: 3.4% to 57.6%). There was a weak tendency towards greater deviations with the increasing number of metastases (Fig. 3b). The deviation between both calculation methods increased when treating small and similarly sized tumor volumes. For example, one case with 3 metastases treated simultaneously (similar tumor volumes: V_1_ = 0.18 ml, V_2_ = 0.14 ml, V_3_ = 1.11 ml) resulted in a deviation of 38%, whereas another case with 3 metastases (one large tumor volume V_1_ = 4.41 ml, two smaller lesions: V_2_ = 0.21 ml, V_3_ = 0.12 ml) resulted in a deviation of only 8%. Index f12 showed no dependence on the number of metastases while using Eq. 5 for the determination of V12 (Fig. 3c) whereas a trend to larger f12 values with the increasing number of metastases was observable when using Eq. 6 (Fig. 3d).

In the following analyses, we used Eq. 5 for V12 estimation of multiple metastases.

For all treatments (40 cases of singular and 40 cases of multiple metastases), f12 ranged from 1.12 (good agreement) up to 4.22 (poor agreement) and was on average 1.91 ± 0.47. Results of index f12 (separated by tumor location and number of metastases) are shown in Table 2.

**Table 2:** Overview of the results of index f12

Index f12	Range	Mean	SD	Number of treatments (singular/multiple)
f12_all data_	1.12–4.22	1.91	0.47	80 (40/40)
f12_multiple, Eq._ 5	1.13–4.22	2.02	0.52	40 (0/40)
f12_singular_	1.12–2.70	1.80	0.39	40 (40/0)
f12_mixed location_	1.13–4.22	2.02	0.63	23 (0/23)
f12_in the parenchyma_	1.12–2.70	1.92	0.34	46 (31/15)
f12_peripheral location_	1.15–2.70	1.63	0.51	11 (9/2)

**Fig. 4:**
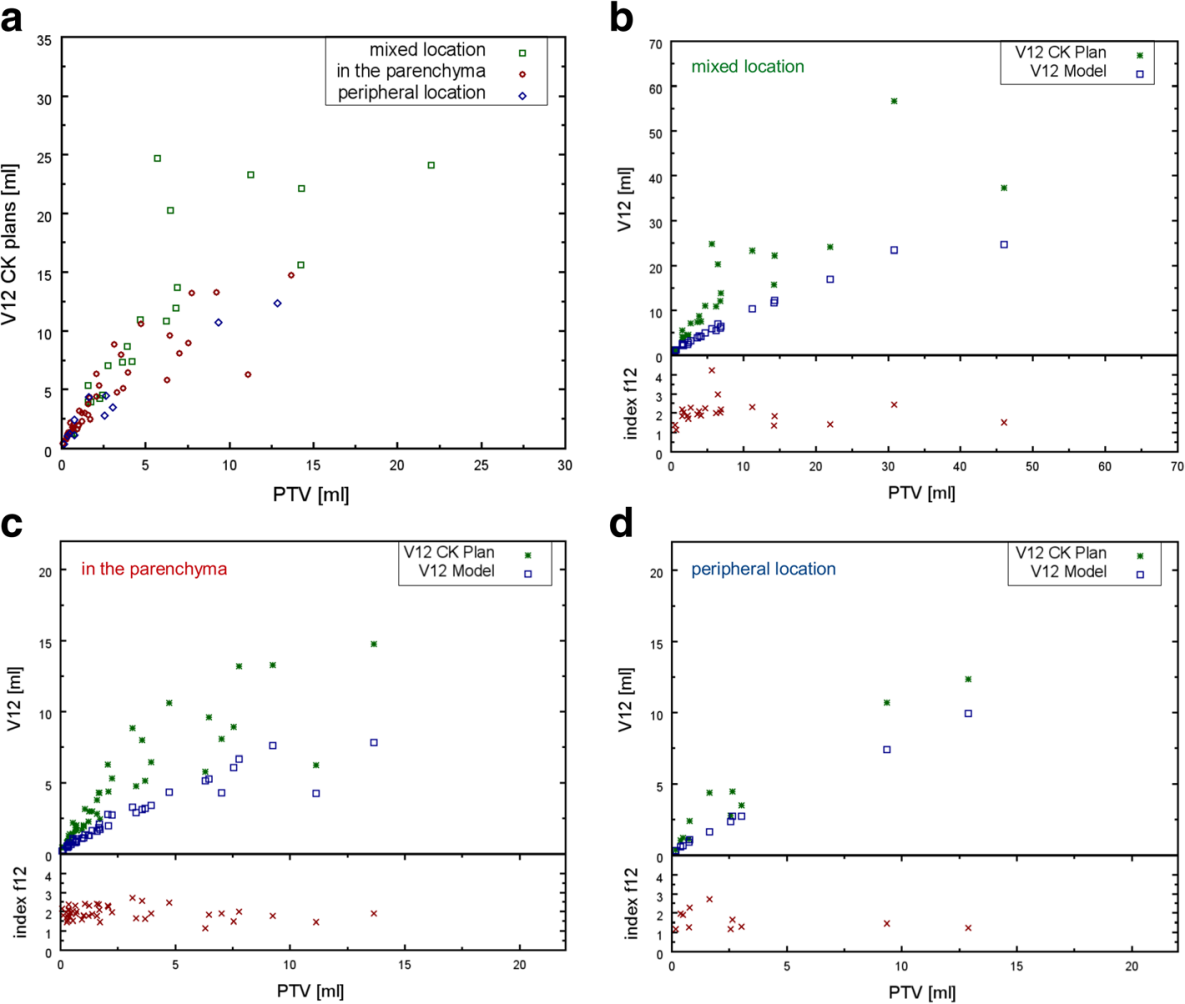
Influence of tumor location on V12 (**a**): Resulting V12 s of the non-isocentric CK plans separated by tumor location for same subset of data as shown in Fig. 3a. Comparison between V12 s of CK plans and model calculation for (**b**): metastases with mixed location, for (**c**): metastases centrally located in the parenchyma and for (**d**): peripheral located metastases

Tumor location showed little influence on the resulting V12 s of the CK plans (Fig. 4). Peripheral tumors (concerns 11 cases) showed less V12 and lower f12 values compared to tumors located in the middle of the parenchyma (Fig. 4c and d). To avoid a bias when evaluating the dependence of index f12 in terms of target volume, nCI, treatment time and total number of MUs, the 11 cases of peripheral tumors were subsequently excluded.

**Fig. 5:**
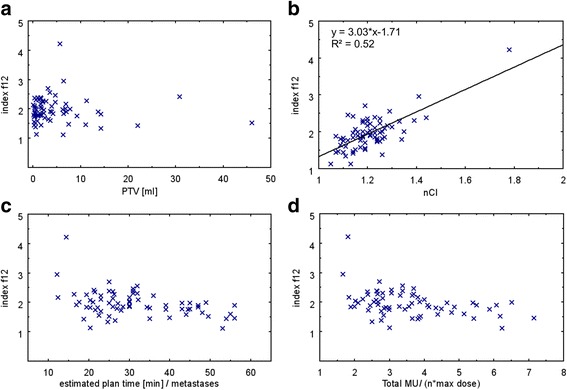
Dependence of index f12 on different factors Relationship between (**a**): index f12 and PTV, **b**: index f12 and nCI, **c**: index f12 and treatment time pro metastasis, **d**: index f12 and total number of MUs (normalized to the number of metastases n multiplied by maximal dose)

Analyzing the dependence of index f12 on the factors mentioned above revealed that f12 is independent of PTV size (Fig. 5a), whereas V12 increases with growing tumor volume (Fig. 3a and Fig. 4a). Better plan conformity (nCI close to one) was associated with smaller f12 values (slope of the linear fit = 3, R^2^ = 0.52, Fig. 5b). For the used patient collective, nCI ranged from 1.05 up to 1.78 (mean = 1.21 ± 0.11). Index f12 was independent of treatment time (Fig. 5c) and of the total number of MUs (Fig. 5d).

## Discussion

Risk analyses of stereotactic radiosurgery have shown that V10 and V12 act as a predictor for brain radionecrosis and thus representing an important parameter for radiotoxicity [11, 12, 18–21]. In our study we present a model that allows the prediction of the lowest achievable V12 at a given tumor volume and prescription dose. The model follows optimum conditions as it assumes a radially symmetrical distribution of the beams from the upper half space, optimal target coverage and plan conformity, as well as spherical target volumes. Considering the underlying assumptions, our model provides a best-case scenario for estimating a minimal-V12. The validity of the model was tested by isocentric plans of a phantom dataset that led to a mean f12 value of 1.2. Deviation from unity might be explained by using voxel data for phantom generation and due to deviations from radially symmetrical dose distribution. Non-isocentric irradiation technique includes the solution space of the isocentric beam setup, but also offers the advantages of sequential optimization which can lead to plan improvements concerning coverage and dosage conformity [6].

Plan quality is characterized by different metrics such as the volumetrically defined coverage, the minimal dose in target volume, dosage conformity and the proportion of the healthy brain volume that is irradiated by more than 10 Gy and 12 Gy, respectively. The planning strategy used in our department aimed at achieving a high coverage (Cov > 98%), an optimal dosage conformity (nCI < 1.3) and a moderate treatment time (30 min-1 h per target). The plan quality was evaluated individually for each case by a decision board (2 physicians, 1 physicist) having regard to patients history, patients age, tumor localization and the number of metastases. In general a high coverage is required to ensure tumor control and to prevent recurrences [27]. Based on our retrospective analyses there were individual cases (tumors located in eloquent brain regions, for instance brainstem metastases or located close to critical structures) in which the physicians accepted a lower coverage or reduced the PD, since a compromise in coverage or dose reduction results in a lower exposure of the normal brain tissue. A reasonable balance between sufficient PD (coverage) to ensure local control and V12 to reduce the risk of radionecrosis is important. The QUANTEC review of Marks et al. recommends keeping V12 lower than 5–10 ml [27]. Our model allows predicting at what tumor size a threshold value of V12 is reached. For example, the group of Minniti et al. [21] recommends considering lesions with V12 > 8.5 ml for hypofractionated SRS to reduce the risk of radionecrosis. Assuming the best-case scenario proposed by our model, V12 of 8.5 ml is achieved at a tumor size of 10.9 ml (tumor diameter of 2.75 cm) when using 65% prescription dose at 20 Gy.

Results of the non-isocentric phantom plans suggested that an expected f12 value of 1.4 on average is possible on the basis of the described sequential optimization strategy. In regard to provide a plan with sufficiently high coverage and since the difference between V12 s of the non-isocentric plans and V12 s of the isocentric plans was low (<1 ml) for small tumors (PTV <10 ml, cf. Fig. 2a), this seems to be an acceptable value of f12.

The application of the model to the clinical cases led to an f12 value of on average 1.9. Index f12 improved with better conformity index nCI and showed no dependence on PTV size or on other plan parameters such as treatment time, total number of MUs, and number of metastases. When keeping the coverage constant at a high level, nCI will be improved if the ratio of PIV to the proportion of PTV covered by the prescribed dose is decreased. Since f12 is defined by the ratio of V12 to the optimum V12 calculated by our model, f12 acts like a conformity index for the 12 Gy isodose level and improves with lower nCI values.

Results of the retrospective analysis showed that f12 values of singular (f12_singular_ = 1.80 ± 0.39) and multiple metastases (f12_multiple_ = 2.02 ± 0.52) were comparable within the standard deviation. Depending on the distance between multiple metastases, dose bridges may occur, which can result in higher V12 s compared to singular metastases. Our model takes the number of metastases into account (Eq. 5), but not the effect of dose bridges. Narayanasamy et al. showed that the total treatment volume is a better predictor of whole brain dose from gamma knife based SRS than the number, shape, or location of the lesions [28] while Sahgal et al. found a significant increase in V12 with increasing number of targets for multitarget SRS [29]. In our study, we found that tumor location shows little influence on the resulting V12 s of the CK plans, while V12 is dependent on the number of metastases and a more precise determination of V12 for multiple metastases is achieved when applying the model to each individual metastasis (cf. Fig. 3).

In order to check whether the proposed f12 index can be used as new planning objective for optimizing the treatment planning of brain metastases, we re-planned some cases of the retrospective analysis (Additional file 1: Table S1). All plans aimed at achieving a high coverage and dosage conformity. The plan comparison showed that for singular metastases lower f12 (V12) can be reached when using a various number of collimators (combination of small and larger ones) and by allowing a longer treatment time which is connected with a larger number of total MUs. Performing additional time reduction optimization steps resulted in increased f12 (V12) values. For multiple metastases a lower f12 (V12) was reached by changing the preconditions of MU-limits (allowing more MU per beam, no conditions for total MU or max. MU per node). A disadvantage of such a strategy is that it can lead to dose fingers in the low-dose range. The CK offers only two collimation systems, a limited number of circular collimators or a variable circular Iris collimator, both providing the same selection of circular fields. The application of a micro-multileaf-collimator (MLC) (for instance the new “InCise MLC”, available for CK M6 system), which enabled the production of irregular-shaped fields, might be a good alternative [30, 31]. Previous planning studies showed a notable reduction in treatment time and in total MU [32, 33].

For assessing plan quality different decision objectives have to be taken into account.

Moreover, the plan quality depends on planning time (testing different beam configurations and collimator settings) and the experience of the treatment planner, and varies between different sites [34]. The proposed f12 index is an instrument which allows an estimation of how far the actual V12 is from the best-case scenario at a given tumor size (independently for small or medium/large tumors) and at a given PD. This information may help to save planning time and to reduce the possible risk of radionecrosis.

The model prediction of V12 and the clinical application of the model have some limitations. The model does not consider the location of the metastases. Theoretically an f12 index less than one is possible for peripheral tumors at the margin to bone or for metastases located close to other structures outside the brain contour because this would lead to an overestimation of the V12-model. Another issue concerns the shape of the tumors; our model assumes spherical metastases that do not reflect the reality. Any deviation from the radially symmetrical tumor shape could influence the dose gradient and lead to larger V12 s. A possible solution to take this aspect into account could be an estimation of the sphericity of the tumors and to implement it in the model calculation.

Our study is focused on the treatment planning of brain metastases with CK, but it is conceivable to transfer the described methods and model assumptions to other systems as well.

## Conclusion

Achieving a minimal V12 for the treatment plans of brain metastases is desirable to lessen the risk of radionecrosis and to spare the healthy surrounding brain. In our study we suggest a new quality parameter (index f12), which is a measure of how far the actual V12 of the plan is from the predicted minimal-V12. Thus, f12 describes the conformity of V12 with the minimal possible V12. It is volume independent and can be used for optimizing treatment plans of singular and multiple brain metastases equally.

## Additional file

Additional file 1: Plan comparison and indicators for low or high f12 indices. (DOCX 29 kb)

## Abbreviations

CK: Cyberknife
GK: Gamma knife
MU: Monitor unit
NCI: new conformity index
OCR: Off-center ratios
PD: Prescription dose
PIV: Prescription isodose volume
PTV: Planning tumor volume
SRS: Stereotactic radiosurgery
V10: Irradiated brain volume (minus tumor volume) at 10 Gy
V12: Irradiated brain volume (minus tumor volume) at 12 Gy
