# Supplementary material for: Radiotoxicity in robotic radiosurgery: proposing a new quality index for optimizing the treatment planning of brain metastases
Source: Radiat Oncol. 2017 Aug 17;12:136. doi: 10.1186/s13014-017-0867-z (PMC5561581; doi:10.1186/s13014-017-0867-z)
Supplement: Additional file 1: — Plan comparison and indicators for low or high f12 indices. (DOCX 29 kb) [file 13014_2017_867_MOESM1_ESM.docx]

**Appendix A: Re-planning and plan comparison of five clinical SRS cases**

**Table A1** Plan comparison and indicators for low or high f12 indices. Values in bold highlight changes in plan results compared to the previous plan.

| **Case # 1** | Num. of met.: 1 | | | | |
| --- | --- | --- | --- | --- | --- |
|  | (PTV = 1.246 ml) | | | | |
| **Specifications of the**  **original plan:** | | **Plan results:** | | | |
| *Num. of collimators* | *Size of collimators (mm)* | *D_min_ (Gy)* | *nCI* | *Cov (%)* | *V12 (ml)* |
| 1 | 10 | 19.93 | 1.18 | 99.71 | 2.97 |
| *max. MU per beam* | *max. MU per node* | *Num. of beams/nodes* | *total MU (10^3^)* | *time (min)* | *f12* |
| 150 | 450 | 86/44 | 9.4 | 30 | 2.31 |
|  | |  | | | |
| **Specifications of the**  **optimized plan #1:** | | **Plan results:** | | | |
| *Num. of collimators* | *Size of collimators (mm)* | *D_min_ (Gy)* | *nCI* | *Cov (%)* | *V12 (ml)* |
| ***2*** | **5+10** | **20.13** | **1.15** | **100** | **2.13** |
| *max. MU per beam* | *max. MU per node* | *Num. of beams/nodes* | *total MU (10^3^)* | *time (min)* | *f12* |
| 150 | 450 | **144/79** | **16.5** | **51** | **1.65** |
|  | |  | | | |
| **optimized plan #2**  **using Time Reduction:** | | **Plan results:** | | | |
| *Num. of collimators* | *Size of collimators (mm)* | *D_min_ (Gy)* | *nCI* | *Cov (%)* | *V12 (ml)* |
| 2 | 5+10 | **20.04** | 1.15 | 100 | **2.38** |
| *max. MU per beam* | *max. MU per node* | *Num. of beams/nodes* | *total MU (10^3^)* | *time (min)* | *f12* |
| 150 | 450 | **116/32** | **11.6** | **39** | **1.85** |
|  | |  | | | |
| **optimized plan #3**  **using additional Time Reduction:** | | **Plan results:** | | | |
| *Num. of collimators* | *Size of collimators (mm)* | *D_min_ (Gy)* | *nCI* | *Cov (%)* | *V12 (ml)* |
| 2 | 5+10 | **19.60** | **1.18** | **99.54** | **2.90** |
| *max. MU per beam* | *max. MU per node* | *Num. of beams/nodes* | *total MU (10^3^)* | *time (min)* | *f12* |
| 150 | 450 | **82/22** | **9.67** | **32** | **2.25** |
|  |  |  |  |  |  |
| **Case # 2** | Num. of met.: 1 | | | | |
|  | (PTV = 1.593 ml) | | | | |
| **Specifications of the**  **original plan:** | | **Plan results:** | | | |
| *Num. of collimators* | *Size of collimators (mm)* | *D_min_ (Gy)* | *nCI* | *Cov (%)* | *V12 (ml)* |
| 1 | 10 | 20.00 | 1.23 | 99.97 | 3.76 |
| *max. MU per beam* | *max. MU per node* | *Num. of beams/nodes* | *total MU (10^3^)* | *time (min)* | *f12* |
| 150 | 450 | 123/60 | 10.4 | 37 | 2.39 |
|  |  |  |  |  |  |
| **Specifications of the**  **optimized plan #1:** | | **Plan results:** | | | |
| *Num. of collimators* | *Size of collimators (mm)* | *D_min_ (Gy)* | *nCI* | *Cov (%)* | *V12 (ml)* |
| **2** | **5+10** | **20.23** | **1.18** | **100** | **2.70** |
| *max. MU per beam* | *max. MU per node* | *Num. of beams/nodes* | *total MU (10^3^)* | *time (min)* | *f12* |
| 150 | 450 | **168/81** | **18.8** | **57** | **1.72** |
|  | |  | | | |
| **optimized plan #2**  **using Time Reduction:** | | **Plan results:** | | | |
| *Num. of collimators* | *Size of collimators (mm)* | *D_min_ (Gy)* | *nCI* | *Cov (%)* | *V12 (ml)* |
| 2 | 5+10 | **20.11** | **1.17** | 100 | 2.95 |
| *max. MU per beam* | *max. MU per node* | *Num. of beams/nodes* | *total MU (10^3^)* | *time (min)* | *f12* |
| 150 | 450 | **164/44** | **14.8** | **50** | **1.87** |
|  |  |  |  |  |  |
| **Case # 3** | Num. of met.: 1 |  |  |  |  |
|  | (PTV = 3.561 ml) |  |  |  |  |
| **Specifications of the**  **original plan:** | | **Plan results:** | | | |
| *Num. of collimators* | *Size of collimators (mm)* | *D_min_ (Gy)* | *nCI* | *Cov (%)* | *V12 (ml)* |
| 1 | 12.5 | 20.49 | 1.29 | 100 | 7.94 |
| *max. MU per beam* | *max. MU per node* | *Num. of beams/nodes* | *total MU (10^3^)* | *time (min)* | *f12* |
| 180 | 540 | 78/44 | 10.4 | 30 | 2.56 |
|  |  |  |  |  |  |
| **Specifications of the**  **optimized plan #1:** | | **Plan results:** | | | |
| *Num. of collimators* | *Size of collimators (mm)* | *D_min_ (Gy)* | *nCI* | *Cov (%)* | *V12 (ml)* |
| **2** | **5+12.5** | **20.08** | **1.12** | 100 | **5.19** |
| *max. MU per beam* | *max. MU per node* | *Num. of beams/nodes* | *total MU (10^3^)* | *time (min)* | *f12* |
| 180 | 540 | **206/44** | **21.4** | **58** | **1.67** |
|  |  |  |  |  |  |
| **Case # 4** | Num. of met.: 3  (total PTV = 4.739 ml) | |  |  |  |
|  |  |  |  |  |  |
| **Specifications of the**  **original plan:** | | **Plan results:** | | | |
| *Num. of collimators* | *Size of collimators (mm)* | *D_min_ (Gy)* | *nCI* | *Cov (%)* | *V12 (ml)* |
| 2 | 5+15 | 19.78 | 1.18 | 99.62 | 10.61 |
| *max. MU per beam* | *max. MU per node* | *Num. of beams/nodes* | *total MU (10^3^)* | *time (min)* | *f12* |
| 180 | 540 | 279/91 | 34.0 | 87 | 2.46 |
|  |  |  |  |  |  |
| **Specifications of the**  **optimized plan #1:** | | **Plan results:** | | | |
| *Num. of collimators* | *Size of collimators (mm)* | *D_min_ (Gy)* | *nCI* | *Cov (%)* | *V12 (ml)* |
| **3** | **5+10+15** | **19.84** | **1.20** | 99.62 | **9.13** |
| *max. MU per beam* | *max. MU per node* | *Num. of beams/nodes* | *total MU (10^3^)* | *time (min)* | *f12* |
| **200** | **600** | **253/106** | **38.0** | **94** | **2.12** |
|  | |  | | | |
| **Specifications of the**  **optimized plan #2:** | | **Plan results:** | | | |
| *Num. of collimators* | *Size of collimators (mm)* | *D_min_ (Gy)* | *nCI* | *Cov (%)* | *V12 (ml)* |
| 3 | 5+10+15 | **19.80** | **1.17** | **99.50** | **8.28** |
| *max. MU per beam* | *max. MU per node* | *Num. of beams/nodes* | *total MU (10^3^)* | *time (min)* | *f12* |
| **500** | **n.a.** | **213/94** | **41.9** | **91** | **1.92** |
|  | |  | | | |
| **Specifications of the**  **optimized plan #3:** | | **Plan results:** | | | |
| *Num. of collimators* | *Size of collimators (mm)* | *D_min_ (Gy)* | *nCI* | *Cov (%)* | *V12 (ml)* |
| **2** | **5+15** | **19.70** | **1.18** | **99.44** | **8.52** |
| *max. MU per beam* | *max. MU per node* | *Num. of beams/nodes* | *total MU (10^3^)* | *time (min)* | *f12* |
| 500 | n.a. | **218/95** | **44.1** | 91 | **1.98** |
|  |  |  |  |  |  |
| **Case # 5** | Num. of met.: 7 |  |  |  |  |
|  | (total PTV = 5.712 ml) | |  |  |  |
| **Specifications of the**  **original plan:** | | **Plan results:** | | | |
| *Num. of collimators* | *Size of collimators (mm)* | *D_min_ (Gy)* | *nCI* | *Cov (%)* | *V12 (ml)* |
| 3 | 5+7.5+12.5 | 18.93 | 1.78 | 99.9 | 24.67 |
| *max. MU per beam* | *max. MU per node* | *Num. of beams/nodes* | *total MU (10^3^)* | *time (min)* | *f12* |
| 150 | n.a. | 295/111 | 38.9 | 101 | 4.22 |
|  |  |  |  |  |  |
| **Specifications of the**  **optimized plan #1:** | | **Plan results:** | | | |
| *Num. of collimators* | *Size of collimators (mm)* | *D_min_ (Gy)* | *nCI* | *Cov (%)* | *V12 (ml)* |
| 3 | 5+7.5+12.5 | **18.58** | **1.47** | **99.34** | **17.81** |
| *max. MU per beam* | *max. MU per node* | *Num. of beams/nodes* | *total MU (10^3^)* | *time (min)* | *f12* |
| **700** | n.a. | **169/79** | **48.2** | **93** | **3.05** |
